# A version of $\kappa$-Miller forcing

**DOI:** 10.1007/s00153-020-00721-y

**Published:** 2020-02-20

**Authors:** Heike Mildenberger, Saharon Shelah

**Affiliations:** 1 Mathematisches Institut, Abteilung für Math. Logik, Albert-Ludwigs-Universität Freiburg, Ernst–Zermelo–Straße 1, 79104 Freiburg im Breisgau, Germany; 2 Institute of Mathematics, The Hebrew University of Jerusalem, Edmond Safra Campus Givat Ram, 9190401 Jerusalem, Israel

**Keywords:** Forcing with higher perfect trees, Primary 03E05, Secondary 03E04, 03E15

## Abstract

We consider a version of $\kappa$-Miller forcing on an uncountable cardinal $\kappa$. We show that under $2^{<\kappa } = \kappa$ this forcing collapses $2^\kappa$ to $\omega$ and adds a $\kappa$-Cohen real. The same holds under the weaker assumptions that ${{\,\mathrm{cf}\,}}(\kappa ) > \omega$, $2^{2^{<\kappa }}= 2^\kappa$, and forcing with $([\kappa ]^\kappa , \subseteq )$ collapses $2^\kappa$ to $\omega$.

## Introduction

Many of the tree forcings on the classical Baire space have various analogues for higher cardinals. Here we are concerned with Miller forcing [4]. In the classical case, a Miller condition is a superperfect subtree of $\omega ^{<\omega }$. The subtree is ordered by the end-extension relation on $\omega ^{<\omega }$. The forcing order is simply $\subseteq$. A tree is superperfect if each node has an extension that has infinitely many immediate tree successors. Such a node is called a splitting node. We can assume that each node has just one direct successor or infinitely many.

For a $\kappa$-version of Miller forcing, superperfectness and splitting are usually interpreted as follows: Above each node $t \in p \subseteq \kappa ^{<\kappa }$ there is a node splitting node *s*. The common interpretation of “*s* is a splitting node of *p*” is:

$$\begin{aligned} \{\alpha \in \kappa \, : \,s \,\hat{} \,\langle \alpha \rangle \in p \} \text{ contains } \text{ a } \text{ club } \text{ subset } \text{ of } \kappa . \end{aligned}$$

In order to gain ($<\kappa$)-closure of the notion of forcing, in addition to the club version of superperfectness one usually requires for conditions that (see, e.g., [2, Section 5.2]) limits of length less than $\kappa$ of splitting nodes be splitting nodes as well.

In this paper we investigate a version of $\kappa$-Miller forcing where the conditions on superperfectness and ($<\kappa$)-closure of splitting nodes are kept and the definition of “*s* is a splitting node of *p*” is weakened to

$$\begin{aligned} |\{\alpha \, : \,s \,\hat{} \,\langle \alpha \rangle \in p\}|=\kappa . \end{aligned}$$

We show: If ${{\,\mathrm{cf}\,}}(\kappa ) > \omega$, ${{\,\mathrm{cf}\,}}(\kappa ) = \kappa$ or ${{\,\mathrm{cf}\,}}(\kappa ) < 2^{{{\,\mathrm{cf}\,}}(\kappa )} \le \kappa$, $2^{2^{<\kappa }} = 2^\kappa$, and there is a $\kappa$-mad family of size $2^\kappa$, then this variant of Miller forcing is related to the forcing $([\kappa ]^\kappa , \subseteq )$ and collapses $2^\kappa$ to $\omega$. In particular, if $\omega<\kappa ^{<\kappa } = \kappa$, then our four premises are fulfilled. Thus we provide some mathematical justification of the customary choice of higher Miller forcing.

Throughout the paper we let $\kappa$ be an uncountable cardinal. We do not make the general assumption that $2^{<\kappa } = \kappa$, nor do we assume that $\kappa$ is regular.

We denote forcing orders in the form $({\mathbb {P}},\le _{{\mathbb {P}}})$ and let $q \le _{\mathbb {P}}p$ mean that *q* is *stronger* than *p*.

If ${{\,\mathrm{dom}\,}}(t), i$ are ordinals, we write $t \,\hat{} \,\langle i\rangle$ for the concatenation of *t* with the singleton function $\{(0,i)\}$, i.e., $t \,\hat{} \,\langle i\rangle =t \cup \{({{\,\mathrm{dom}\,}}(t), i)\}$. For cardinals $\kappa$, $\lambda$, we write ${}^{<\lambda } \kappa$ for the set of functions $f :\alpha \rightarrow \kappa$ for some $\alpha < \lambda$. For $s, t \in \kappa ^{<\lambda }$ we write $s \trianglelefteq t$ if $s = t \restriction {{\,\mathrm{dom}\,}}(s)$, and the corresponding strict order is written as $\triangleleft$. The domain $\alpha$ of *f* is also called the length of *f*. The set of subsets of $\kappa$ of size $\kappa$ is denoted by $[\kappa ]^\kappa$.

### Definition 1.1

1. ${\mathbb {Q}}_\kappa ^1$ is the forcing $([\kappa ]^\kappa , \subseteq )$.
2. ${\mathbb {Q}}_\kappa ^2$ is the following version of $\kappa$-Miller forcing: Conditions are trees $T \subseteq {}^{\kappa > } \kappa$ that are $\kappa$ *superperfect*: for each $s \in T$ there is $s \trianglelefteq t$ such that *t* is a $\kappa$-splitting node of *T*. A node $t \in T$ is called a $\kappa$-*splitting node* if
  $$\begin{aligned} {{\,\mathrm{set}\,}}_p(t) = \{\alpha < \kappa \, : \,t \,\hat{} \,\langle \alpha \rangle \in T\} \end{aligned}$$
  has size $\kappa$. The set of splitting nodes of *T* is denoted by ${{\,\mathrm{spl}\,}}(T)$.
  We furthermore require for $p \in {\mathbb {Q}}^2_\kappa$ that the limit of an $\triangleleft$-increasing sequence of length less than $\kappa$ of $\kappa$-splitting nodes is a $\kappa$-splitting node if it has length less than $\kappa$.
  For $p,q \in {\mathbb {Q}}_\kappa ^2$ we write $q \le _{{\mathbb {Q}}^2_\kappa } p$ if $q \subseteq p$. So subtrees are stronger conditions.
3. For $p \in {\mathbb {Q}}_\kappa ^2$ and $\eta \in p$ we let ${{\,\mathrm{suc}\,}}_p(s) = \{t \in {}^{\kappa >} \kappa \, : \,(\exists \alpha < \kappa ) (t= s \,\hat{} \,\langle \alpha \rangle \in p)\}$.
4. Let $s \in p \in {\mathbb {Q}}^2_\kappa$. We let $p^{\langle s \rangle } = \{t\in p \, : \,t \trianglelefteq s \vee s \trianglelefteq t\}$.
5. For $a, b \subseteq \kappa$ we write $a \subseteq ^*_\kappa b$ if $|a \setminus b|< \kappa$.

Each of the two forcing orders ${\mathbb {P}}$ has a weakest element, denoted by $1_{{\mathbb {P}}}$. Namely, ${\mathbb {Q}}^1_\kappa$ has as a weakest element $1_{{\mathbb {Q}}^1_\kappa } = \kappa$, and ${\mathbb {Q}}^2_\kappa$ has as a weakest element the full tree ${}^{\kappa >} \kappa$. We write ${\mathbb {P}}\Vdash \varphi$ if the weakest condition forces $\varphi$.

## Results about ${\mathbb {Q}}^1_\kappa$

In this section we consider ${\mathbb {Q}}^1_\kappa$. The purpose is to provide standardised ${\mathbb {Q}}^1_\kappa$-names for collapses. Later these particular ${\mathbb {Q}}^1_\kappa$-names shall be translated to ${\mathbb {Q}}^2_\kappa$-names.

### Definition 2.1

A family ${\mathcal {A}}\subseteq [\kappa ]^\kappa$ is called a $\kappa$-almost disjoint family if for $A \ne B \in {\mathcal {A}}$, $|A \cap B|<\kappa$.

### Observation 2.2

If $2^{<\kappa } = \kappa$, there is a $\kappa$-ad family ${\mathcal {A}}\subseteq [\kappa ]^\kappa$ of size $2^\kappa$.

### Proof

We let $f :{}^{\kappa >} 2 \rightarrow \kappa$ be an injection. We assign to each branch *b* of ${}^{\kappa >}2$ a set $a_b=\{f(s) \, : \,s \in b\}$. The resulting family $\{a_b \, : \,b \text{ branch } \text{ of } {}^{\kappa >} 2\}$ is $\kappa$-ad. $\square$

### Observation 2.3

If ${\mathbb {Q}}^1_\kappa$ collapses $2^\kappa$ to $\omega$, then there is a $\kappa$-ad family of size $2^\kappa$.

### Proof

${\mathbb {Q}}^1_\kappa$ cannot have the $2^\kappa$-c.c. Hence there is an antichain of size $2^\kappa$. Since $p \perp _{{\mathbb {Q}}^1_\kappa } q$ means $|p \cap q| < \kappa$, the antichain is a $\kappa$-ad family. $\square$

We will apply the following result for $\chi = 2^\kappa$.

### Theorem 2.4

[5, Theorem 0.5] Suppose that there is an antichain in ${\mathbb {Q}}^1_\kappa$ of size $\chi$. Then the following holds.

1. Forcing with ${\mathbb {Q}}^1_\kappa$ collapses $\chi$ to $\aleph _0$ if $\aleph _0 < {{\,\mathrm{cf}\,}}(\kappa ) = \kappa$ or if $\aleph _0< {{\,\mathrm{cf}\,}}(\kappa ) < 2^{{{\,\mathrm{cf}\,}}(\kappa )} \le \kappa$.
2. Forcing with ${\mathbb {Q}}^1_\kappa$ collapses $\chi$ to $\aleph _1$ in the case of $\aleph _0 = {{\,\mathrm{cf}\,}}(\kappa )<\kappa$.

Now we start defining tree structures from ${\mathbb {Q}}^1_\kappa$-names for collapsing functions. Those trees will later be used to define dense suborders $Q_{{\mathcal {T}}}$ of ${\mathbb {Q}}^2_\kappa$. The idea of $Q_{{\mathcal {T}}}$ is that the sets ${{\,\mathrm{set}\,}}_p(t)$, $t \in {{\,\mathrm{spl}\,}}(p)$, for $p \in Q_{{\mathcal {T}}}$ will be sufficiently strong ${\mathbb {Q}}^1_\kappa$ conditions.

### Lemma 2.5

Suppose that ${\mathbb {Q}}_\kappa ^1$ collapses $2^\kappa$ to $\omega$. Then there is a ${\mathbb {Q}}^1_\kappa$-name  for a surjection, and there is a labelled tree ${{\mathcal {T}}}= \langle (a_\eta , n_\eta , \varrho _\eta ) \, : \,\eta \in {}^{\omega >}(2^\kappa )\rangle$ with the following properties

- $a_{\langle \rangle } = \kappa$ and for any $\eta \in {}^{\omega >}(2^\kappa )$, $a_\eta \in [\kappa ]^\kappa$.
- $$\eta _1 \triangleleft \eta _2$$ implies $$a_{\eta _1} \supseteq a_{\eta _2}$$.
- $$n_\eta \in [{{\,\mathrm{dom}\,}}(\eta )+1, \omega )$$.
- If $a \in [\kappa ]^\kappa$ then there is some $\eta \in {}^{\omega >} (2^\kappa )$ such that $a \supseteq a_\eta$.
- If $\eta \,\hat{} \,\langle \beta \rangle \in T$ then $a_{\eta \,\hat{} \,\langle \beta \rangle }$ forces  for some $\varrho _{\eta \,\hat{} \,\langle \beta \rangle } \in {}^{n_\eta } (2^\kappa )$, such that the $\varrho _{\eta \,\hat{} \,\langle \beta \rangle }$, $\beta \in 2^\kappa$, are pairwise different. Hence for any $\eta \in {}^{\omega >}(2^\kappa )$, the family $\{a_{\eta \,\hat{} \,\langle \alpha \rangle } \, : \,\alpha < 2^\kappa \}$ is a $\kappa$-ad family in $[a_\eta ]^\kappa$.

### Proof

Let  be a ${\mathbb {Q}}^1_\kappa$-name such that  is onto. For $\alpha < 2^\kappa$ let $AP_\alpha$ be the set of objects ${\bar{m}}$ satisfying

$$(*)_1$$
(1.1)  $${\bar{m}} = (T,{\bar{a}},{\bar{n}},{\bar{\varrho }}) = (T_{{\bar{m}}}, {\bar{a}}_{{\bar{m}}}, {\bar{n}}_{{\bar{m}}}, {\bar{\varrho }}_{{\bar{m}}})$$.
  (1.2)  *T* is a subtree of $({}^{\omega >} (2^\kappa ), \triangleleft )$ of cardinality $\le |\alpha | + \kappa$ and $\langle \rangle \in T$.
  (1.3)  ${\bar{a}} = \langle a_\eta \, : \,\eta \in T\rangle$ fulfils $\eta \triangleleft \nu \rightarrow a_\nu \subseteq a_\eta$ and $a_{\langle \rangle } = \kappa$ and $a_\eta \in [\kappa ]^{\kappa }$.
  (1.4)  ${\bar{n}} = \langle n_\eta \, : \,\eta \in T \rangle$ fulfils ${{\,\mathrm{dom}\,}}(\varrho _{\eta \,\hat{} \,\langle \beta \rangle }) = n_\eta > {{\,\mathrm{dom}\,}}(\eta )$ for any $\eta \,\hat{} \,\langle \beta \rangle \in T$.
  (1.5)  If $\eta \,\hat{} \,\langle \beta \rangle \in T$, then $a_{\eta \,\hat{} \,\langle \beta \rangle }$ forces a value to , called $\varrho _{\eta \,\hat{} \,\langle \beta \rangle }$, and for $\beta \ne \gamma$ we have $\varrho _{\eta \,\hat{} \,\langle \beta \rangle } \ne \varrho _{\eta \,\hat{} \,\langle \gamma \rangle }$. Hence for any $\eta \,\hat{} \,\langle \beta \rangle$, $\eta \,\hat{} \,\langle \gamma \rangle \in T_{{\bar{m}}}$, $\beta \ne \gamma$ implies $a_{\eta \,\hat{} \,\langle \beta \rangle } \cap a_{\eta \,\hat{} \,\langle \gamma \rangle } \in [\kappa ]^{<\kappa }$.
  (1.6)  For $\eta \in T_{{\bar{m}}}$, we let  and require that the latter has cardinality $2^\kappa$.

In the next items we state some properties of $AP_\alpha$ that are derived from $(*)_1$.

$$(*)_2$$
$AP = \bigcup \{AP_\alpha \, : \,\alpha < 2^\kappa \}$ is ordered naturally by $\le _{AP}$, which means end extension.
$$(*)_3$$
(a)  $AP_\alpha$ is not empty and increasing in $\alpha$.
  (b)  For infinite $\alpha$, $AP_\alpha$ is closed under unions of increasing sequences of length $< |\alpha |^+$.
$$(*)_4$$
Let $\gamma < 2^\kappa$. If ${\bar{m}} \in AP_\gamma$ and $\eta \in T_{{\bar{m}}}$ and $\eta \,\hat{} \,\langle \alpha \rangle \not \in T_{{\bar{m}}}$ then there is ${\bar{m}}' \in AP_\gamma$ such that ${\bar{m}} \le _{AP} {\bar{m}}'$ and $T_{{\bar{m}}'} = T_{{\bar{m}}} \cup \{\eta \,\hat{} \,\langle \alpha \rangle \}$.
  Proof: For $\eta \in T_{{\bar{m}}}$,  whereas
  $$\begin{aligned} \Lambda _\eta = \{ \varrho _{\eta \,\hat{} \,\langle \beta \rangle } \restriction n_\eta \, : \,\beta \in 2^\kappa \wedge \eta \,\hat{} \,\langle \beta \rangle \in T_{{\bar{m}}}\} \end{aligned}$$
  is of size $\le |T_{{\bar{m}}}| \le |\gamma | + \kappa$. Hence we can choose $\varrho _*\in {\mathcal {U}}\setminus \Lambda _\eta$ and $b_*\in [a_\eta ]^\kappa$ such that . We let $\varrho _{\eta \,\hat{} \,\langle \alpha \rangle } = \varrho _*$. Since $b_*$ forces a value of $\tau \restriction n_\eta$ that is incompatible with the one forced by $a_{\eta \,\hat{} \,\langle \beta \rangle }$ for any $\eta \,\hat{} \,\langle \beta \rangle \in T_{{\bar{m}}}$, the set $b_*$ is $\kappa$-almost disjoint from $a_{\eta \,\hat{} \,\langle \beta \rangle }$ for any $\eta \,\hat{} \,\langle \beta \rangle \in T_{{\bar{m}}}$. We take $b_*= a_{{\bar{m}}', \eta \,\hat{} \,\langle \alpha \rangle } \subseteq a_{{\bar{m}},\eta }$.

Since ${{\,\mathrm{cf}\,}}(2^\kappa ) > \aleph _0$ and sincethere is an *n* such thathas cardinality $2^\kappa$. We take the minimal one and let it be $n_{\eta \,\hat{} \,\langle \alpha \rangle }$.

$$(*)_5$$
If ${\bar{m}} \in AP_\alpha$ and $a \in [\kappa ]^\kappa$ then there is some ${\bar{m}}' \ge {\bar{m}}$, such that there is $\eta \in T_{{\bar{m}}'}$ with $a_{{\bar{m}}',\eta } \subseteq a$.
  Let  i.e.  This set has cardinality $2^\kappa$ because  is onto. We take *n* minimal such that  has size $2^\kappa$. We let
  $$\begin{aligned} {{\,\mathrm{set}\,}}_n^+({\bar{m}}) = \{ \varrho _{\eta } \, : \,\eta \in T_{{\bar{m}}}, {{\,\mathrm{dom}\,}}(\varrho _\eta ) \ge n\}. \end{aligned}$$
  Clearly $|{{\,\mathrm{set}\,}}_n^+({\bar{m}})| \le |T_{{\bar{m}}}| \le |\gamma | + \kappa$. Thus we can take $\varrho _a \in {\mathcal {U}}_{a,n}$ that is incompatible with every element of ${{\,\mathrm{set}\,}}^+_n({\bar{m}})$. We take some $b_a \in [a]^\kappa$ such that . The set
  $$\begin{aligned} \Lambda _a = \{ \eta \in T_{{\bar{m}}} \, : \,b_a \subseteq ^*_\kappa a_\eta \} \end{aligned}$$
  is $\triangleleft$-linearly ordered by $(*)_1$ clauses 1.3 and 1.5 and $\langle \rangle \in \Lambda _a$. Since $b_a$ does not pin down , $\Lambda _a$ has a $\triangleleft$-maximal member $\eta _a$. Now we take $\alpha _*= \min \{\beta \, : \,\eta _a \,\hat{} \,\langle \beta \rangle \not \in T_{{\bar{m}}}\}$. For any $\eta _a \,\hat{} \,\langle \beta \rangle \in T_{{\bar{m}}}$ we have $\varrho _{\eta _a \,\hat{} \,\langle \beta \rangle }$ and $\varrho _a$ are incompatible, and hence $a_{\eta _a \,\hat{} \,\langle \beta \rangle } \cap b_a \in [\kappa ]^{<\kappa }$. Now we choose $b_a^1 \in [b_a]^\kappa$ and $\varrho ^*_a$ such that and ${{\,\mathrm{dom}\,}}(\varrho ^*_a) \ge n_{{\bar{m}},\eta _a}> {{\,\mathrm{dom}\,}}(\eta _a)$.
  We let
  $$\begin{aligned} T_{{\bar{m}}'}= & {} T_{{\bar{m}}} \cup \{\eta _a \,\hat{} \,\langle \alpha _*\rangle \},\\ a_{\eta _a \,\hat{} \,\langle \alpha _*\rangle }= & {} b^1_a, \end{aligned}$$
  We let $n_{\eta _a \,\hat{} \,\langle \alpha _*\rangle }$ be the minimal *n* such that $|\mathrm{Pos}(b_a^1,n)| \ge 2^\kappa$. So $(*)_5$ holds.

Now we are ready to construct ${\mathcal {T}}$ as in the statement of the lemma. We do this by recursion on $\alpha \le 2^{\kappa }$. First we enumerate $[\kappa ]^\kappa$ as $\langle c_\alpha \, : \,\alpha < 2^\kappa \rangle$, and we enumerate ${}^{\omega >} (2^\kappa )$ as $\langle \eta _\alpha \, : \,\alpha < 2^\kappa \rangle$ such that $\eta _\alpha \triangleleft \eta _\beta$ implies $\alpha < \beta$. We choose an increasing sequence ${\bar{m}}_\alpha$ by induction on $\alpha <2^\kappa$. We start with the tree $\{\langle \rangle \}$, $a_{\langle \rangle }= \kappa$, $\varrho _{\langle \rangle } = \emptyset$, $n_{\langle \rangle }$ be minimal such that $|\mathrm{Pos}(\kappa ,n)| = 2^\kappa$. In the odd successor steps we take ${\bar{m}}_{2\alpha +1} \ge _{AP} {\bar{m}}_\alpha$ so that $a_\eta \subseteq c_\alpha$ for some $\eta \in T_{2\alpha +1}$. This is done according to $(*)_5$. In the even successor steps we take ${\bar{m}}_{2\alpha +2} \ge _{AP} {\bar{m}}_{2\alpha +1}$ such that $\eta _\alpha \in T_{2\alpha +2}$. Since all initial segments of $\eta _\alpha$ appeared among the $\eta _\beta$, $\beta < \alpha$, ${\bar{m}}_{2\alpha +2}$ is found according to $(*)_4$. In the limit steps we take unions. Then ${{\mathcal {T}}}$ that is given by the last three components of ${\bar{m}}_{2^\kappa }$ has properties (a) to (e). $\square$

Since  is not in $\mathbf {V}$, for any ${\mathcal {T}}$ as in Lemma 2.5, for any $f \in {}^\omega (2^\kappa ) \cap {\mathbf {V}}$, the branch $\langle (a_{f\restriction m},n_{f\restriction m}, \varrho _{f \restriction m}) \, : \,m \in \omega \rangle$ of ${\mathcal {T}}$ has a no $\subseteq ^*_\kappa$-lower bound for its first coordinate.

## Transfer to ${\mathbb {Q}}^2_\kappa$

In this section we use the tree ${\mathcal {T}}$ from Lemma 2.5 for finding ${\mathbb {Q}}^2_\kappa$-names. First we establish a dense subforcing $Q_{{\mathcal {T}}}$ of ${\mathbb {Q}}^2_\kappa$. Then we construct $Q_{{\mathcal {T}}}$-names that are based on a ${\mathbb {Q}}^1_\kappa$-name of a collapse and on the equation $2^{2^{<\kappa }} = 2^\kappa$.

### Definition 3.1

Let $\mu , \lambda$ be cardinals. For $\nu , \nu ' \in {}^{\lambda >}\mu$ we write $\nu \perp \nu '$ if $\nu \not \trianglelefteq \nu '$ and $\nu ' \not \trianglelefteq \nu$.

Typical pairs $(\lambda , \mu )$ are $(\omega , 2^\kappa )$ and $(\kappa ,\kappa )$.

An important tool for the analysis of ${\mathbb {Q}}^2_\kappa$ is the following particular kind of fusion sequence $\langle p_\alpha \, : \,\alpha< \kappa ^{<\kappa } \rangle$ in ${\mathbb {Q}}^2_\kappa$. Since we do not suppose $\kappa ^{<\kappa } = \kappa$, a fusion sequence can be longer than $\kappa$. An important property is that for each $\nu \in {}^{\kappa >}\kappa$ there is at most one $\alpha< \kappa ^{<\kappa }$ such that ${{\,\mathrm{set}\,}}_{p_\alpha }(\nu ) \supsetneq {{\,\mathrm{set}\,}}_{p_{\alpha +1}}(\nu )$.

### Lemma 3.2

Let $\langle \nu _\alpha \, : \,\alpha< \kappa ^{<\kappa } \rangle$ be an injective enumeration of $\kappa ^{<\kappa }$ such that

3.1 $$\begin{aligned} \nu _\alpha \triangleleft \nu _\beta \rightarrow \alpha < \beta . \end{aligned}$$

Let $\langle p_\alpha , \nu _\alpha , c_\alpha \, : \,\alpha< \kappa ^{<\kappa } \rangle$ be a sequence such that for any $\alpha \le \lambda$ the following holds:

- $$p _0 \in {\mathbb {Q}}^2_\kappa $$.
- If $\alpha = \beta +1< \kappa ^{<\kappa }$ and $\nu _\beta \in {{\,\mathrm{spl}\,}}(p_\beta )$, then
  $$\begin{aligned} \begin{aligned}&c_\beta \in [{{\,\mathrm{suc}\,}}_{p_\beta }(\nu _\beta )]^\kappa \text{ and } \\&p_{\alpha }= p_\beta (\nu _\beta , c_\beta ) := \bigcup \{p_\beta ^{\langle \nu _\beta \,\hat{} \,\langle i \rangle \rangle } \, : \,i \in c_\beta \} \cup \bigcup \{p_\beta ^{\langle \eta \rangle } \, : \,\eta \not \trianglelefteq \nu _\beta \wedge \nu _\beta \not \trianglelefteq \eta \} \end{aligned} \end{aligned}$$
- If $\alpha = \beta +1< \kappa ^{<\kappa }$ and $\nu _\beta \not \in {{\,\mathrm{spl}\,}}(p_\beta )$ then $p_\alpha = p_\beta$.
- $$p_\alpha = \bigcap \{p_\beta \, : \,\beta <\alpha \}$$ for limit $$\alpha \le \kappa ^{<\kappa }$$.

Then for any $\lambda \le \kappa ^{<\kappa }$, $p_\lambda \in {\mathbb {Q}}^2_\kappa$ and $\forall \beta < \lambda$, $p_\lambda \le _{{\mathbb {Q}}^2_\kappa } p_\beta$.

### Proof

We go by induction on $\lambda$. The case $\lambda =0$ and the successor steps are obvious. So we assume that $\lambda \le \kappa ^{<\kappa }$ is a limit ordinal and $p_\alpha \in {\mathbb {Q}}^2_\kappa$ for $\alpha < \lambda$. Since $\emptyset \in p_\lambda$, $p_\lambda$ is not empty, and $p_\lambda$ clearly is a tree. Let $t \in p_\lambda$. We show that there is $t' \trianglerighteq t$ that is a splitting node in $p_\lambda$.

We fix the smallest $\alpha$ such that $\nu _\alpha \trianglerighteq _{p_0} t$ is a splitting node in $p_0$. Then in $p_0$ there are no splitting nodes in $\{s \, : \,t \trianglelefteq s \triangleleft \nu _\alpha \}$. Hence $\nu _\alpha \in {{\,\mathrm{spl}\,}}(p_{\beta })$ for any $\beta \in [0,\lambda ]$.

Now we show that the limit of splitting nodes in $p_\lambda$ is a splitting node. Let $\gamma < \lambda$ and let $\langle \nu ^{i} \, : \,i < \gamma \rangle$ be an $\triangleleft$-increasing sequence of splitting nodes of $p_\lambda$ with union $\nu \in \kappa ^{<\kappa }$. Then $\nu$ is a splitting node of each $p_\alpha$, $\alpha < \lambda$, and also in $p_\lambda$ since $\langle {{\,\mathrm{set}\,}}_{p_\alpha }(\nu ) \, : \,\alpha < \lambda \rangle$ has at most two entries and their intersection has size $\kappa$. $\square$

We use yet another, richer type of fusion sequence.

### Definition 3.3

Let $p \in {\mathbb {Q}}^2_\kappa$ and let $\nu \in {{\,\mathrm{spl}\,}}(p)$.

1. We say $\eta$ is *the shortest splitting node above* $\nu$ *in* *p* and write $\eta = {{\,\mathrm{sucspl}\,}}_p(\nu )$ if $\eta$ is the shortest splitting node in *p* such that $\eta \supseteq \nu$. Equality is allowed and occurs if $\nu$ is a splitting node.
2. We say $F \subseteq p$ is *the front of next splitting nodes above* $\nu$ *in* *p*, if
  $$\begin{aligned} F = \{ \eta ' \in {{\,\mathrm{spl}\,}}(p) \, : \,\exists (\eta \in {{\,\mathrm{suc}\,}}_p(\nu ))(\eta ' = {{\,\mathrm{sucspl}\,}}_p(\eta ))\}. \end{aligned}$$

### Lemma 3.4

Let $\langle \nu _\alpha \, : \,\alpha< \kappa ^{<\kappa } \rangle$ be an injective enumeration of $\kappa ^{<\kappa }$ such that

3.2 $$\begin{aligned} \nu _\alpha \triangleleft \nu _\beta \rightarrow \alpha < \beta . \end{aligned}$$

Let $\langle p_\alpha , \nu _\alpha , c_\alpha , F_{\alpha } \, : \,\alpha< \kappa ^{<\kappa } \rangle$ be a sequence such that for any $\alpha \le \lambda$ the following holds:

- $$p _0 \in {\mathbb {Q}}^2_\kappa $$.
- If $\alpha = \beta +1< \kappa ^{<\kappa }$ and $\nu _\beta \in sp(p_\beta )$, then $c_\beta \in [{{\,\mathrm{suc}\,}}_{p_\beta }(\nu _\beta )]^\kappa$, $F_{\beta }$ contains for each $i \in c_\beta$ exactly one $\eta \in {{\,\mathrm{spl}\,}}(p_\beta ^{\langle \nu _\beta \,\hat{} \,\langle i \rangle \rangle })$, and
  $$\begin{aligned} \begin{aligned} p_{\alpha }= p_\beta (\nu _\beta , c_\beta , F_{\beta }) :=&\bigcup \{p_\beta ^{\langle \eta \rangle } \, : \,i \in c_\beta , \eta \in F_{\beta } \}\\&\cup \bigcup \{p_\beta ^{\langle \eta \rangle } \, : \,\eta \not \trianglelefteq \nu _\beta \wedge \nu _\beta \not \trianglelefteq \eta \}. \end{aligned} \end{aligned}$$
  Note that this implies that $F_\beta$ is the front of next splitting nodes of $p_\alpha$ above $\nu _\beta$.
- If $\alpha = \beta +1< \kappa ^{<\kappa }$ and $\nu _\beta \not \in {{\,\mathrm{spl}\,}}(p_\beta )$ then $p_\alpha = p_\beta$.
- $$p_\alpha = \bigcap \{p_\beta \, : \,\beta <\alpha \}$$ for limit $$\alpha \le \kappa ^{<\kappa }$$.

Then for any $\lambda \le \kappa ^{<\kappa }$, $p_\lambda \in {\mathbb {Q}}^2_\kappa$ and $\forall \beta < \lambda$, $p_\lambda \le _{{\mathbb {Q}}^2_\kappa } p_\beta$.

### Proof

We go by induction on $\lambda$. The case $\lambda =0$ and the successor steps are obvious. So we assume that $\lambda \le \kappa ^{<\kappa }$ is a limit ordinal and $p_\alpha \in {\mathbb {Q}}^2_\kappa$ for $\alpha < \lambda$. Since $\emptyset \in p_\lambda$, $p_\lambda$ is not empty, and $p_\lambda$ clearly is a tree. Let $t \in p_\lambda$. We show that there is $t' \trianglerighteq t$ that is a splitting node in $p_\lambda$.

We fix the smallest $\alpha$ such that $\nu _\alpha \trianglerighteq _{p_0} t$ is a splitting node in $p_0$. Then in $p_0$ there are no splitting nodes in $\{s \, : \,t \trianglelefteq s \triangleleft \nu _\alpha \}$. Hence $\nu _\alpha \in {{\,\mathrm{spl}\,}}(p_{\beta })$ for any $\beta \in [0,\lambda ]$.

Now we show that the limit of splitting nodes in $p_\lambda$ is a splitting node. Let $\gamma < \lambda$ and let $\langle \nu ^{i} \, : \,i < \gamma \rangle$ be an $\triangleleft$-increasing sequence of splitting nodes of $p_\lambda$ with union $\nu \in \kappa ^{<\kappa }$. Then $\nu$ is a splitting node of each $p_\alpha$, $\alpha < \lambda$, and also in $p_\lambda$ since $\langle {{\,\mathrm{set}\,}}_{p_\alpha }(\nu ) \, : \,\alpha < \lambda \rangle$ has at most two entries and their intersection has size $\kappa$. $\square$

In the special case $F_\beta = \{\nu _\beta \,\hat{} \,\langle j \rangle \, : \,j \in c_\beta \}$, the construction of Lemma 3.4 coincides with the simpler construction from Lemma 3.2.

### Definition 3.5

We assume ${\mathbb {Q}}^1_\kappa$ collapses $2^\kappa$ to $\omega$. Let  and ${{\mathcal {T}}} = \langle (a_\eta ,n_\eta ,\varrho ) \, : \,\eta \in {}^{\omega >}(2^\kappa )\rangle$ be as in Lemma 2.5. Now let $Q_{{\mathcal {T}}}$ be the set of $\kappa$-Miller trees *p* such that for every $\nu \in {{\,\mathrm{spl}\,}}(p)$ there is $\eta _{p,\nu } = \eta _\nu \in {}^{\omega >} (2^\kappa )$ such that

3.3 $$\begin{aligned} {{\,\mathrm{set}\,}}_p(\nu ) = \{ \varepsilon \in \kappa \, : \,\nu \,\hat{} \,\langle \varepsilon \rangle \in p \} = a_{\eta _\nu }. \end{aligned}$$

By the properties of ${\mathcal {T}}$, the node $\eta _{p,\nu }$ is unique.

### Lemma 3.6

Assume that ${\mathbb {Q}}^1_\kappa$ collapses $2^\kappa$ to $\omega$, let ${\mathcal {T}}$ be chosen as in Lemma 2.5, and let $Q_{{\mathcal {T}}}$ be defined from ${\mathcal {T}}$ as above. Then $Q_{{\mathcal {T}}}$ is dense in ${\mathbb {Q}}^2_\kappa$.

### Proof

Let $p_0 = T \in {\mathbb {Q}}^2_\kappa$. Let $\langle \nu _\alpha \, : \,\alpha<\kappa ^{<\kappa } \rangle$ be an injective enumeration of $\kappa ^{<\kappa }$ with property (3.1). We now define fusion sequence $\langle p_\alpha ,\nu _\alpha , c_\alpha \, : \,\alpha \le \kappa ^\kappa \rangle$ according to the pattern in Lemma 3.2 in order to find $p_{\kappa ^{<\kappa }} \ge T$ such that $p_{\kappa ^{<\kappa }} \in Q_{{\mathcal {T}}}$.

Suppose that $p_\alpha$ and $\nu _\alpha$ are given. If $\nu _\alpha$ is not in $p_\alpha$ or is not a splitting node in $p_\alpha$, then we let $p_{\alpha +1} = p_\alpha$. If $\nu _\alpha \in {{\,\mathrm{spl}\,}}(p_\alpha )$, then according to Lemma 2.5 clause (d) there is $\eta \in {}^{\omega >} (2^\kappa )$ such that ${{\,\mathrm{suc}\,}}_{p_\alpha }(\nu _\alpha ) \supseteq a_\eta$. We choose such an $\eta$ of minimal length and call it $\eta (\alpha )$.

Then we strengthen $p_\alpha$ to

3.4 $$\begin{aligned} \begin{aligned} p_{\alpha +1} =&\bigcup \{p_\alpha ^{\langle \nu ' \rangle } \, : \,\nu ' = \nu _\alpha \,\hat{} \,\langle i \rangle \wedge i \in a_{\eta (\alpha )}\} \cup \\&\bigcup \{p_\alpha ^{\langle \eta \rangle } \, : \,\eta \not \trianglelefteq \nu _\alpha \wedge \nu _\alpha \not \trianglelefteq \eta \} .\end{aligned} \end{aligned}$$

Now we have that

$$\begin{aligned} \eta _{p_{\alpha +1},\nu _\alpha }=\eta (\alpha ), c_\alpha = a_{\eta (\alpha )}. \end{aligned}$$

For limit ordinals $\lambda \le \kappa ^{<\kappa }$, we let $p_\lambda = \bigcap \{p_\beta \, : \,\beta < \lambda \}$. Since the sequence $\langle p_\alpha , \nu _\alpha , c_\alpha \, : \,\alpha \le \kappa ^{<\kappa } \rangle$ matches the pattern in Lemma 3.2, we have $p_{\kappa ^{<\kappa }} \in {\mathbb {Q}}^2_\kappa$. By construction, for any $\alpha< \kappa ^{<\kappa }$ for any $\delta \in [\alpha +1,\kappa ^{<\kappa })$, $\nu _\alpha \in {{\,\mathrm{spl}\,}}(p_\delta )$ implies

$$\begin{aligned} {{\,\mathrm{set}\,}}_{p_{\alpha +1}}(\nu _\alpha ) = {{\,\mathrm{set}\,}}_{p_\delta }(\nu _\alpha )=a_{\eta (\alpha )}. \end{aligned}$$

Hence the condition $p=p_{\kappa ^{<\kappa }}$ fulfils Equation (3.3) in its splitting node $\nu _\alpha$ with witness $\eta _{p,\nu _\alpha }= \eta (\alpha )$. Since all nodes are enumerated, we have $p_{\kappa ^{<\kappa }} \in Q_{{\mathcal {T}}}$. $\square$

We use only the inclusion ${{\,\mathrm{set}\,}}_p(\nu ) \subseteq a_{\eta _\nu }$ from Definition 3.5.

### Definition 3.7

We assume that ${\mathbb {Q}}^1_\kappa$ collapses $2^\kappa$ to $\omega$ and the ${{\mathcal {T}}}$ is as in Lemma 2.5. For $T \in Q_{{\mathcal {T}}}$ and a splitting node $\nu$ of *T* we set $\varrho _{T,\nu } := \varrho _{\eta _{T,\nu }} \in {}^{\omega >} (2^\kappa )$. Recall $\eta _{T,\nu }$ is defined in Def. 3.5, and $\varrho$ is a component of ${{\mathcal {T}}}$.

For $p \in Q_{{\mathcal {T}}}$, the relation $\nu \trianglelefteq \nu ' \in p$ does neither imply $\eta _{\nu } \trianglelefteq \eta _{\nu '}$ nor $\varrho _\nu \trianglelefteq \varrho _{\nu '}$. However, $\eta _\nu \triangleleft \eta _{\nu '}$ implies $a_{\eta _\nu } \supset a_{\eta _{\nu '}}$ and $\varrho _\nu \triangleleft \varrho _{\nu '}$.

### Observation 3.8

We assume that ${\mathbb {Q}}^1_\kappa$ collapses $2^\kappa$ to $\omega$. Let $p_1, p_2 \in Q_{{\mathcal {T}}}$. If $p_1 \le _{{\mathbb {Q}}^2_\kappa } p_2$ then for $\nu \in {{\,\mathrm{spl}\,}}(p_2)$ we have $\nu \in {{\,\mathrm{spl}\,}}(p_1)$ and $\varrho _{p_1,\nu } \trianglelefteq \varrho _{p_2,\nu }$.

We introduce dense sets:

### Definition 3.9

We assume that ${\mathbb {Q}}^1_\kappa$ collapses $2^\kappa$ to $\omega$. Let $n \in \omega$.

$$\begin{aligned} D_n = \bigl \{p \in Q_{{\mathcal {T}}} \, : \,(\forall \nu \in {{\,\mathrm{spl}\,}}(p)) ({{\,\mathrm{dom}\,}}(\varrho _{p,\nu }) > n)\}. \end{aligned}$$

$D_n$ is open dense in $Q_{{\mathcal {T}}}$ and the intersection of the $D_n$ is empty.

Recall, by Lemma 3.6 we can work with the dense subforcing $Q_{{\mathcal {T}}}$ of ${\mathbb {Q}}^2_\kappa$. The following technical lemma is the next step of a transformation of a ${\mathbb {Q}}^1_\kappa$-name of a surjection from $\omega$ onto $2^\kappa$ into a $Q_{{\mathcal {T}}}$-name of such a surjection. The coordinate ${\bar{\gamma }}_\alpha$ and the clauses (d), (e), (f) are used for a counting argument in the induction steps. Later, only the coordinates $p_\alpha$, $n_\alpha$, and clauses (a), (b), (c) and Remark 3.11 will be used.

### Lemma 3.10

We assume that ${\mathbb {Q}}^1_\kappa$ collapses $2^\kappa$ to $\omega$, ${{\,\mathrm{cf}\,}}(\kappa ) > \omega$ and $2^{(\kappa ^{<\kappa })} = 2^\kappa$. Let $\langle T_{\alpha } \, : \,\alpha < 2^\kappa \rangle$ enumerate ${\mathbb {Q}}^2_\kappa$ such that each Miller tree appears $2^\kappa$ times. There is $\langle (p_\alpha , n_\alpha , {\bar{\gamma }}_\alpha ) \, : \,\alpha < 2^\kappa \rangle$ such that

- $$n_\alpha < \omega $$,
- $$p_\alpha \in D_{n_\alpha } \subseteq Q_{{\mathcal {T}}}$$ and $$p_\alpha \ge T_\alpha $$.
- If $\beta < \alpha$ and $n_\beta \ge n_\alpha$ then $p_\beta \perp p_\alpha$.
- $${\bar{\gamma }}_\alpha = \langle \gamma _{\alpha ,\nu } \, : \,\nu \in {{\,\mathrm{spl}\,}}(p_\alpha )\rangle $$.
- $$(\forall \nu \in {{\,\mathrm{spl}\,}}(p_\alpha )) (a_{\eta _{p_\alpha ,\nu }} \Vdash _{{\mathbb {Q}}^1_\kappa } \gamma _{\alpha ,\nu } \in {{\,\mathrm{range}\,}}(\varrho _{p_\alpha ,\nu }))$$.
- $$\gamma _{\alpha ,\nu } \in 2^\kappa \setminus W_{<\alpha ,\nu }$$ with
  $$\begin{aligned} W_{<\alpha ,\nu } = \bigcup \{{{\,\mathrm{range}\,}}(\varrho _{p_\beta ,\nu }) \, : \,\beta < \alpha , \nu \in {{\,\mathrm{spl}\,}}(p_\beta )\}. \end{aligned}$$

### Proof

Assume that $\langle (p_\beta , n_\beta , {\bar{\gamma }}_\beta ) \, : \,\beta < \alpha \rangle$ has been defined and we are to define $(p_\alpha ,n_\alpha , {\bar{\gamma }}_\alpha )$. Note that the $p_\beta$ need not be increasing in strength.

$$(\oplus )_1$$
The choice of the $a_\eta$ in Lemma 2.5 and the choice $Q_{{\mathcal {T}}}$ and of $\eta _{p_\beta ,\nu }$ for $\nu \in {{\,\mathrm{spl}\,}}(p_\beta )$, $\beta < \alpha$, imply that the set $W_{<\alpha ,\nu }$ is well defined and of cardinality $\le |\alpha | + \aleph _0 < 2^\kappa$. Hence we can choose $\gamma _{\alpha ,\nu } \in 2^\kappa \setminus W_{<\alpha ,\nu }$.
$$(\oplus )_2$$
With the fusion Lemma 3.2 we choose $q_\alpha \ge T_\alpha$, $q_\alpha \in Q_{{\mathcal {T}}}$, such that
  $$\begin{aligned} (\forall \nu \in {{\,\mathrm{spl}\,}}(q_\alpha ))(a_{\eta _{q_\alpha ,\nu }} \Vdash _{{\mathbb {Q}}^1_\kappa } \gamma _{\alpha ,\nu } \in {{\,\mathrm{range}\,}}(\varrho _{q_\alpha ,\nu })). \end{aligned}$$
$$(\oplus )_3$$
Let $q \in {\mathbb {Q}}^2_\kappa$. For $n \in \omega$ and $\nu \in {{\,\mathrm{spl}\,}}(q)$ we let
  $$\begin{aligned} {\mathcal {U}}_{\alpha ,\nu ,n}(q) = \{ \beta < \alpha \, : \,n_\beta = n , \nu \in {{\,\mathrm{spl}\,}}(p_\beta ) \wedge |{{\,\mathrm{set}\,}}_{q}(\nu ) \cap {{\,\mathrm{set}\,}}_{p_\beta }(\nu ) | = \kappa \}. \\ {\mathcal {U}}_{\alpha , \nu }(q) = \bigcup \{{\mathcal {U}}_{\alpha ,\nu ,n}(q) \, : \,n \in \omega \}. \end{aligned}$$
$$(\oplus )_4$$
(a) If $n \in \omega$ and $\nu \in {{\,\mathrm{spl}\,}}(q_\alpha )$ then
  $$\begin{aligned} \beta \in {\mathcal {U}}_{\alpha ,\nu }(q_\alpha ) \rightarrow \varrho _{p_\beta ,\nu } \trianglelefteq \varrho _{q_\alpha ,\nu }. \end{aligned}$$
  This is seen as follows. We let $a= {{\,\mathrm{set}\,}}_{p_\beta }(\nu ) \cap {{\,\mathrm{set}\,}}_{q_\alpha }(\nu )$. Since $\beta \in {\mathcal {U}}_{\alpha ,\nu }(q_\alpha )$, $a \in [\kappa ]^\kappa$. Clearly . So either $\varrho _{p_\beta ,\nu } \triangleleft \varrho _{q_\alpha ,\nu }$ or $\varrho _{p_\beta ,\nu } \trianglerighteq \varrho _{q_\alpha ,\nu }$. However, since $\gamma _{\alpha ,\nu } \in {{\,\mathrm{range}\,}}(\varrho _{q_\alpha ,\nu }) \setminus W_{<\alpha ,\nu }$, only $\varrho _{q_\alpha ,\nu } \triangleright \varrho _{p_\beta ,\nu }$ is possible.
  (b) So for $\nu \in {{\,\mathrm{spl}\,}}(q_\alpha )$, the set $\{ \varrho _{p_\beta ,\nu } \, : \,\beta \in {\mathcal {U}}_{\alpha ,\nu }(q_\alpha )\}$ has at most ${{\,\mathrm{dom}\,}}(\varrho _{q_\alpha ,\nu })$ elements.
  (c) The assigment $\beta \mapsto \varrho _{p_\beta ,\nu }$ is is defined between ${\mathcal {U}}_{\alpha ,\nu }(q_\alpha )$ and $\{ \varrho _{p_\beta ,\nu } \, : \,\beta \in {\mathcal {U}}_{\alpha ,\nu }(q_\alpha )\}$. According to properties (e) and (f) in the induction hypothesis, the assigment is injective, and hence
  $$|{\mathcal {U}}_{\alpha ,\nu }(q_\alpha ) |\le {{\,\mathrm{dom}\,}}(\varrho _{q_\alpha ,\nu })$$.
  (d) We state for further use that ${\mathcal {U}}_{\alpha ,\nu }(q_\alpha )$ is finite and for any $q \le q_\alpha$, ${\mathcal {U}}_{\alpha ,\nu }(q) \subseteq {\mathcal {U}}_{\alpha ,\nu }(q_\alpha )$.
$$(\oplus )_5$$
We look at the cone above $q_\alpha$ and show:
  3.5 $$\begin{aligned} \begin{aligned}&(\forall q \ge q_\alpha )(\forall \nu \in {{\,\mathrm{spl}\,}}(q)) (\exists r_{\alpha ,\nu }\le _{{\mathbb {Q}}^2_\kappa } q)\\&(\exists c \in [{{\,\mathrm{set}\,}}_q(\nu )]^\kappa )(\exists F \subseteq \{\eta \in {{\,\mathrm{spl}\,}}(q) \, : \,\eta \triangleright \nu \})\\&\bigl (r_{\alpha ,\nu } = q(\nu ,c,F) \wedge (\forall \beta \in {\mathcal {U}}_{\alpha ,\nu }(q_\alpha )) (r^{\langle \nu \rangle }_{\alpha ,\nu } \perp p_\beta ^{\langle \nu \rangle } \vee p_\beta ^{\langle \nu \rangle } \ge r^{\langle \nu \rangle }_{\alpha ,\nu })\bigr ). \end{aligned} \end{aligned}$$

How do we find $r_{\alpha ,\nu }= r_{\alpha ,\nu }(q)$? Given $q \le _{{\mathbb {Q}}^2_\kappa } q_\alpha$, $\nu \in {{\,\mathrm{spl}\,}}(q)$ we enumerate ${\mathcal {U}}_{\alpha ,\nu }(q_\alpha )$ as $\beta _0$, ..., $\beta _{k-1}$. We let $r_{0} = q$ and by induction on $i \le k$ we define $r_i$, increasing in strength, with $\nu \in {{\,\mathrm{spl}\,}}(r_i)$ and $c_i = {{\,\mathrm{set}\,}}_{r_i}(\nu )$. Thus the $c_i$ are $\subseteq$-decreasing sets of size $\kappa$. Given $r_i$, we distinguish cases:

First case: $\beta _i \not \in {\mathcal {U}}_{\alpha ,\nu }(r_i)$. Then there is $c_{i+1} \in [{{\,\mathrm{set}\,}}_{r_i}(\nu )]^\kappa$, $c_{i+1} \cap {{\,\mathrm{set}\,}}_{p_{\beta _i}}(\nu ) = \emptyset$. We let $r_{i+1} = r_i(\nu , c_{i+1})$ and thus have $r_{i+1}^{\langle \nu \rangle } \perp p_{\beta _i}$.

Second case: $\beta _i \in {\mathcal {U}}_{\alpha ,\nu }(r_i)$. We let

$$\begin{aligned} c_i = \{j \in {{\,\mathrm{set}\,}}_{r_i}(\nu ) \, : \,r_i^{\langle \nu \,\hat{} \,\langle j \rangle \rangle } \le p_{\beta _i}^{\langle \nu \,\hat{} \,\langle j \rangle \rangle }\} \cup \{j \in {{\,\mathrm{set}\,}}_{r_i}(\nu ) \, : \,r_i^{\langle \nu \,\hat{} \,\langle j \rangle \rangle } \not \le p_{\beta _i}^{\langle \nu \,\hat{} \,\langle j \rangle \rangle }\}. \end{aligned}$$

If $c_{i,1} = \{j \in {{\,\mathrm{set}\,}}_{r_i}(\nu ) \, : \,r_i^{\langle \nu \,\hat{} \,\langle j \rangle \rangle } \le p_{\beta _i}^{\langle \nu \,\hat{} \,\langle j \rangle \rangle }\}$ has size $\kappa$, then we let $c_{i+1} = c_{1,i}$ and $r_{i+1} = r_i(\nu ,c_{i+1})$ and thus get $r_{i+1}^{\langle \nu \rangle } \ge p_{\beta _i}$.

If $|c_{i,1}|< \kappa$, then $c_{i,2} = \{j \in {{\,\mathrm{set}\,}}_{r_i}(\nu ) \, : \,r_i^{\langle \nu \,\hat{} \,\langle j \rangle \rangle } \not \le p_{\beta _i}^{\langle \nu \,\hat{} \,\langle j \rangle \rangle }\}$ has size $\kappa$, and we let $c_{i+1} = c_{i,2}$. For $j \in c_{i+1}$, $r_i^{\langle \nu \,\hat{} \,\langle j \rangle \rangle } \not \le p_{\beta _i}^{\langle \nu \,\hat{} \,\langle j\rangle \rangle }$. Thus we can find a node in the $r_i^{\langle \nu \,\hat{} \,\langle j \rangle \rangle }\setminus p_{\beta _i}^{\langle \nu \,\hat{} \,\langle j\rangle \rangle }$ and above this node we find a splitting node of $r_i$. We take this latter splitting node into $r_{i+1}$ as the direct successor splitting node to $\nu \,\hat{} \,\langle j \rangle$. Doing so for every $j \in c_{i+1}$ we get $F_{\nu ,i}$, a front strictly above $\nu$ in $r_{i+1}= r_i(\nu ,c_{i+1}, F_{\nu ,i})$. Again we get $r_{i+1}^{\langle \nu \rangle } \perp p_{\beta _i}$.

In the end we let $r_{\alpha ,\nu } = r_k$. There is a front *F* that contains for each $j \in c_k$ the shortest splitting node of $r_k$ above $\nu \,\hat{} \,\langle j \rangle$. Thus we have $r_k = r_{\alpha ,\nu }= q(\nu ,c_k, F)$ and $r_{\alpha ,\nu }$ fulfils (3.5).

$$(\oplus )_6$$
Now we use $(\oplus )_5$ iteratively along all $\nu \in \kappa ^{<\kappa }$ to find a fusion sequence $\langle r_{\alpha ,\nu },\nu ,c_\nu , F_{\nu } \, : \,\nu< \kappa ^{<\kappa }\rangle$ with starting point $q_\alpha = r_{0,\nu _0}$. In this sequence, $r_{\alpha , \nu }$ is chosen as $r_{\alpha ,\nu }(q)$ in $\oplus _5$ for $q = \bigcap _{\beta < \alpha } r_\beta$, if $\nu \in {{\,\mathrm{spl}\,}}(q)$. If $\nu \not \in {{\,\mathrm{spl}\,}}(q)$, then $r_{\alpha ,\nu } = q$. Then we apply the fusion Lemma 3.4 and get an lower bound $r_\alpha$ of $r_{\alpha ,\nu }$, $\nu \in {}^{\kappa >}\kappa$. Note $r_\alpha ^{\langle \nu \rangle } \perp p_\beta$ iff $r_\alpha ^{\langle \nu \rangle } \perp p_\beta ^{\langle \nu \rangle }$ and $r_\alpha ^{\langle \nu \rangle } \le p_\beta$ iff $r_\alpha ^{\langle \nu \rangle } \le p_\beta ^{\langle \nu \rangle }$. Hence $r_\alpha \ge q_\alpha$ and
  $$\begin{aligned} (\forall \nu \in {{\,\mathrm{spl}\,}}(r_\alpha ))( \forall \beta \in {\mathcal {U}}_{\alpha ,\nu }(q_\alpha )) (r_\alpha ^{\langle \nu \rangle } \perp p_\beta \vee p_\beta \ge r_\alpha ^{\langle \nu \rangle }). \end{aligned}$$
$$(\oplus )_7$$
Finally we choose $n_\alpha$ and $p_\alpha$. There are *k* and $\nu$ such that $n < \omega$ and $\nu \in {{\,\mathrm{spl}\,}}(r_\alpha )$ such that $p_\alpha = r_\alpha ^{\langle \nu \rangle }$ fulfils
  $$\begin{aligned} (\forall \beta < \alpha )( n_\beta \ge k \rightarrow p_\alpha \perp p_\beta ). \end{aligned}$$
  Proof of existence. By induction on $k \in \omega$ we try to find $\langle \nu _k, \beta _k \, : \,k \in \omega \rangle$ such that
  (a) $$\nu _k \in {{\,\mathrm{spl}\,}}(r_\alpha )$$,
  (b) $$\nu _k \triangleleft \nu _m$$ for $$k <m$$,
  (c) $$\beta _k < \alpha $$ and $$n_{\beta _k} \ge k$$ and $$r_\alpha ^{\langle \nu _k \rangle } \le p_{\beta _k}$$.
  If we succeed, then $\nu _*=\bigcup \{\nu _k \, : \,k \in \omega \} = \nu ^* \in {{\,\mathrm{spl}\,}}(r_\alpha )$ by Definition 1.1 (2). Here we use that ${{\,\mathrm{cf}\,}}(\kappa ) > \omega$. Hence  This is a contradiction.

So there is a smallest *k* such that $\nu _k$ cannot be defined. We let $n_\alpha = k$. We let $p_\alpha$ be a strengthening of $r_\alpha ^{\langle \nu _{k-1} \rangle }$ such that $p_\alpha \in D_{n_\alpha }$. For finding such a strengthening we again invoke the fusion Lemma 3.2.

We show that $p_\alpha \perp p_\beta$ for $\beta < \alpha$ with $n_\beta \ge k$. Otherwise, having arrived at $r_\alpha ^{\langle \nu _{k-1}\rangle }$ we find some $\beta _k , \alpha$ such that $n_{\beta _k} \ge k$ and $r_\alpha ^{\langle \nu _{k-1}\rangle }$ is compatible with $p_{\beta _k}$. Then we can prolong $\nu _{k-1}$ to a splitting node $\nu _k \in {{\,\mathrm{spl}\,}}(p_{\beta _k}) \cap {{\,\mathrm{spl}\,}}(r_{\alpha })$. By the choice of $r_\alpha$ the latter implies that $r_{\alpha }^{\langle \nu _k \rangle } \le p_{\beta _k}$. However, now we would have found $\nu _k, \beta _k$ as required in contradiction to the choice of *k*. $\square$

### Remark 3.11

Conditions (a) to (c) of Lemma 3.10 yield: For any $k <\omega$,

$$\begin{aligned} \{p_\alpha \, : \,n_\alpha \ge k\} \text{ is } \text{ dense } \text{ in } {\mathbb {Q}}^2_\kappa . \end{aligned}$$

### Proof

Let *k* and *p* be given. There is $\alpha _0$ such that $T_{\alpha _0} \in D_0$ and $T_{\alpha _0} \le _{{\mathbb {Q}}^2_\kappa } p$. Then $p_{\alpha _0} \le T_{\alpha _0}$ and $n_{\alpha _0}\ge 0$. Then there is $\alpha _1> \alpha _0$ such that $T_{\alpha _1} \le _{{\mathbb {Q}}^2_\kappa } p_{\alpha _0}$. Then $p_{\alpha _1} \le T_{\alpha _1}$ and hence by condition (c), $n_{\alpha _1} >n_{\alpha _0}\ge 0$. We can can repeat the argument $k-1$ times. $\square$

Now we drop the component ${\bar{\gamma }}_\alpha$ from a sequence $\langle p_\alpha , n_\alpha , {\bar{\gamma }}_\alpha \, : \,\alpha < 2^\kappa \rangle$ given by Lemma 3.10. Then we get a sequence with properties (a), (b), and a weakening (c) with the property stated in the remark. This sequence, combined with $2^{(2^{<\kappa })} = 2^\kappa$, allows to define a ${\mathbb {Q}}^2_\kappa$-name of a collapse.

### Lemma 3.12

We assume that ${\mathbb {Q}}^1_\kappa$ collapses $2^\kappa$ to $\omega$, ${{\,\mathrm{cf}\,}}(\kappa ) > \omega$ and $2^{(2^{<\kappa })} = 2^\kappa$. Let $\langle T_{\alpha } \, : \,\alpha < 2^\kappa \rangle$ enumerate all Miller trees that such each tree appears $2^\kappa$ times. Assume that $\langle (p_\alpha , n_\alpha ) \, : \,\alpha < 2^\kappa \rangle$ are such that

- $$n_\alpha < \omega $$,
- $$p_\alpha \in D_{n_\alpha }\subseteq Q_{{\mathcal {T}}}$$ and $$p_\alpha \ge T_\alpha $$,
- if $\beta < \alpha$ and $n_\beta = n_\alpha$ then $p_\beta \perp p_\alpha$,
- for any $k \in \omega$, $\{p_\alpha \, : \,n_\alpha \ge k\}$ is dense in ${\mathbb {Q}}^2_\kappa$.

Then there is a ${\mathbb {Q}}^2_\kappa$-name  for a surjection of $\omega$ onto $2^\kappa$.

### Proof

Let *G* be a ${\mathbb {Q}}^2_\kappa$-generic filter over ${\mathbf {V}}$. We define , a ${\mathbb {Q}}^2_\kappa$-name by  if $p_\alpha \in G$ and $n_\alpha = n$. The name  is a name of a function by (c). By (d), the domain of  is forced to be infinite. For any $p \in {\mathbb {Q}}^2_\kappa$ we let $U_p = \{ \alpha \, : \,T_\alpha = p\}$. $U_p$ is of size $2^\kappa$, in particular for $\alpha \in 2^\kappa$ we have $|U_{p_\alpha }|= 2^\kappa$ and $U_{p_\alpha }$ contains the antichain $\{p_{\delta (\alpha ,i)} \, : \,i < 2^\kappa \}$. Hence there is $f :2^\kappa \rightarrow 2^\kappa$ in $\mathbf {V}[G]$ such that for any $\gamma , \alpha \in 2^\kappa$ there is $\beta = \delta (\alpha ,\gamma ) \in U_{p_\alpha }$ for some function $\delta :2^\kappa \times 2^\kappa \rightarrow 2^\kappa$ with $f(\beta ) = \gamma$ forced by $p_{\delta (\alpha ,\gamma )}$. We letNext we showSuppose $p \in Q_{{\mathcal {T}}}$ and $\gamma < 2^\kappa$ are given. By construction the sequence $\{p_\beta \, : \,\beta < 2^\kappa \}$ is dense. Let $p \le p_\alpha$. Then there is $\beta =\delta (\alpha , \gamma ) \in U_{p_\alpha }$ $p_{\delta (\alpha ,\gamma )} \le p_\gamma$, with $f(\alpha ) = \gamma$. However, $\delta (\alpha ,\gamma )=\beta \in U_{p_\alpha }$ means $T_\beta = p_\alpha \ge p_\beta$ by construction. By the definition of , , so . $\square$

So we can sum up:

### Theorem 3.13

We assume that ${\mathbb {Q}}^1_\kappa$ collapses $2^\kappa$ to $\omega$ and ${{\,\mathrm{cf}\,}}(\kappa ) > \omega$ and $2^{(\kappa ^{<\kappa })} = 2^\kappa$. Then the forcing with ${\mathbb {Q}}_\kappa ^2$ collapses $2^\kappa$ to $\aleph _0$.

## $\kappa$-Cohen reals and the Levy collapse

Many $\kappa$-tree forcings add a $\kappa$-Cohen real, sometimes even if their $\omega$-version does not add a Cohen real. Also our forcing ${\mathbb {Q}}^2_\kappa$ is of this kind. Classical Miller forcing preserves *P*-points and hence does not add a Cohen real. In this section we show that under the above conditions, ${\mathbb {Q}}^\kappa _2$ add a $\kappa$-Cohen real and is equivalent to the Levy collapse of $2^\kappa$ to $\aleph _0$.

### Lemma 4.1

If ${\mathbb {Q}}^2_\kappa$ collapses $2^\kappa$ to $\aleph _0$, ${{\,\mathrm{cf}\,}}(\kappa ) > \aleph _0$, and $2^{2^{<\kappa }} = 2^\kappa$, then ${\mathbb {Q}}_\kappa ^2$ adds a $\kappa$-Cohen real.

### Proof

Let *G* be ${\mathbb {Q}}^2_\kappa$-generic over $\mathbf {V}$. Let $f :\omega \rightarrow 2^{<\kappa }$ be a function in $\mathbf{V}[G]$, such that $(\forall \eta \in 2^{<\kappa })(\exists ^\infty k f(k) = \eta )$. Such a function exists since $2^{< \kappa } \le 2^\kappa$.

Since $2^{2^{<\kappa }} = 2^\kappa$, we can enumerate all antichains in ${\mathbb {C}}(\kappa )$ in $\alpha _*\le 2^\kappa$ many steps. In $\mathbf {V}[G]$, $\alpha _*$ is countable. We list it as $\langle \alpha _n \, : \,n < \omega \rangle$. Now we choose $\eta _n \in {\mathbb {C}}(\kappa )^{\mathbf {V}}$ by induction on *n* in $\mathbf {V}[G]$: $\eta _0 = \emptyset$. Given $\eta _n$ we choose $k_n$ such that $f(k_n) = \eta _n$ and then we choose $\eta _{n+1} \trianglerighteq \eta _n$, such that $\eta _{n+1} \in I_{\alpha _n}$. Then $\{\eta \, : \,(\exists n < \omega ) (\eta \trianglelefteq f(k_n))\}$ is a ${\mathbb {C}}(\kappa )$-generic filter over $\mathbf {V}$ and it exists in *V*[*G*], since it is definable from $\{f(k_n) \, : \,n < \omega \}$. $\square$

Two forcings ${\mathbb {P}}_1$, ${\mathbb {P}}_2$ are said to be equivalent if their regular open algebras ${{\,\mathrm{RO}\,}}({\mathbb {P}}_i)$ coincide (for a definition of the regular open algebra of a poset, see, e.g., [3, Corollary 14.12]). Some forcings are characterised up to equivalence just by their size and their collapsing behaviour.

### Lemma 4.2

[3, Lemma 26.7]. Let $(Q, <)$ be a notion of forcing such that $|Q| = \lambda > \aleph _0$ and such that *Q* collapses $\lambda$ onto $\aleph _0$ , i.e.,

$$\begin{aligned} 1_{Q} \Vdash _Q |\check{\lambda }| = \aleph _0. \end{aligned}$$

Then $$\mathrm{RO}(Q) = {{\,\mathrm{Levy}\,}}(\aleph _0,\lambda )$$.

### Lemma 4.3

If ${\mathbb {Q}}_\kappa ^1$ collapses $2^\kappa$ to $\aleph _0$, then ${\mathbb {Q}}_\kappa ^1$ is equivalent of ${{\,\mathrm{Levy}\,}}(\aleph _0, 2^\kappa )$.

### Proof

${\mathbb {Q}}^1_\kappa$ has size $2^\kappa$. Hence Lemma 4.2 yields $\mathrm{RO}({\mathbb {Q}}^1_\kappa )= \mathrm{Levy}(\aleph _0,2^\kappa )$. $\square$

### Definition 4.4

A Boolean algebra is $(\theta ,\lambda )$-*nowhere distributive* if there are antichains ${\bar{p}}^\varepsilon = \langle p^\varepsilon _\alpha \, : \,\alpha < \alpha _\varepsilon \rangle$ of ${\mathbb {P}}$ for $\varepsilon < \theta$ such that for every $p \in {\mathbb {P}}$ for some $\varepsilon < \theta$

$$\begin{aligned} |\{\alpha < \alpha _\varepsilon \, : \,p \not \perp p^\varepsilon _\alpha \}| \ge \lambda . \end{aligned}$$

### Definition 4.5

Let *B* be a Boolean algebra. We write $B^+ = B \setminus \{0\}$. A subset $D \subseteq B^+$ is called *dense* if $(\forall b \in B^+)(\exists d \in D)(d \le b)$. The *density* of a Boolean algebra *B* is the least size of a dense subset of *B*. A Boolean algebra *B* has uniform density if for every $a \in B^ +$, $B \restriction a$ has the same density. The *density* of a forcing order $({\mathbb {P}},\le _{{\mathbb {P}}})$ is the density of the regular open algebra ${{\,\mathrm{RO}\,}}({\mathbb {P}})$.

### Lemma 4.6

[1, Theorem 1.15] Let $\theta < \lambda$ be regular cardinals.

1. Suppose that ${\mathbb {P}}$ has the following properties (a) to (c).
  - ${\mathbb {P}}$ is a $(\theta ,\lambda )$-nowhere distributive forcing notion,
  - $${\mathbb {P}}$$ has density $$\lambda $$,
  - in case $\theta > \aleph _0$, ${\mathbb {P}}$ has a $\theta$-complete dense subset *S*. The latter means: $(\forall B \in [S]^{<\theta })(\exists s\in S)(\forall b \in B)(b \le _{{\mathbb {P}}} s)$.

*Then* ${\mathbb {P}}$ is equivalent to ${{\,\mathrm{Levy}\,}}(\theta ,\lambda )$.

(2) Under (a) and (b) ${\mathbb {P}}$ collapses $\lambda$ to $\theta$ (and may or may not collapse $\lambda$ to $\aleph _0$).

### Proposition 4.7

If there is a $\kappa$-mad family of size $2^\kappa$ the forcing ${\mathbb {Q}}^1_\kappa$ is $(\aleph _0,2^\kappa )$-nowhere distributive.

### Proof

Lemma 2.5 gives ${\mathcal {T}}$ such that ${\bar{p}}^n = \{ a_\eta \, : \,\eta \in {}^n (2^\kappa )\}$, $n \in \omega$, witnesses $(\aleph _0,2^\kappa )$-nowhere distributivity. $\square$

By Lemma 4.2 and Theorem 3.13 we get:

### Proposition 4.8

If ${\mathbb {Q}}^1_\kappa$ collapses $2^\kappa$ to $\aleph _0$, ${{\,\mathrm{cf}\,}}(\kappa ) > \aleph _0$ and $2^{(\kappa ^{<\kappa })} = 2^\kappa$ then ${\mathbb {Q}}^2_\kappa$ is equivalent to $\mathrm{Levy}(\aleph _0,2^\kappa )$.
